
JOURNAL INFORMATION
==============================
NLM Title Abbreviation: Biospektrum (Heidelb)
Journal ID: BIOspektrum (Heidelb.)
NLM Journal ID: 9615685

Biospektrum

ISSN: 0947-0867
EISSN: 1868-6249

ARTICLE INFORMATION
==============================
PMCID: PMC7318724
PMID: 32834537
DOI: 10.1007/s12268-020-1410-6
Article ID: 1410
Article version: 1
Subjects: Editorial

Corona und Forschungsförderung: Unterstützung und Impulse im Zeichen der Pandemie

Becker Katja praesidentin@dfg.de http://www.dfg.de

grid.424150.6 0000 0001 1957 9997 Deutsche Forschungsgemeinschaft (DFG), Kennedyallee 40, D-53175 Bonn, Deutschland
Electronic publication date: 2020 Jun 26
Print publication date: 2020
Volume: 26
Issue: 4
First page: 355
Last page: 355
Received 2020 Jun 5; Accepted 2020 Jun 9
Copyright: © Die Autorin 2020
License: Open Access: Dieser Artikel wird unter der Creative Commons Namensnennung 4.0 International Lizenz veröffentlicht, welche die Nutzung, Vervielfältigung, Bearbeitung, Verbreitung und Wiedergabe in jeglichem Medium und Format erlaubt, sofern Sie den/die ursprünglichen Autor(en) und die Quelle ordnungsgemäß nennen, einen Link zur Creative Commons Lizenz beifügen und angeben, ob Änderungen vorgenommen wurden. Die in diesem Artikel enthaltenen Bilder und sonstiges Drittmaterial unterliegen ebenfalls der genannten Creative Commons Lizenz, sofern sich aus der Abbildungslegende nichts anderes ergibt. Sofern das betreffende Material nicht unter der genannten Creative Commons Lizenz steht und die betreffende Handlung nicht nach gesetzlichen Vorschriften erlaubt ist, ist für die oben aufgeführten Weiterverwendungen des Materials die Einwilligung des jeweiligen Rechteinhabers einzuholen. Weitere Details zur Lizenz entnehmen Sie bitte der Lizenzinformation auf http://creativecommons.org/licenses/by/4.0/deed.de.
License URL: https://creativecommons.org/licenses/by/4.0/

issue-copyright-statement: © Springer-Verlag GmbH Deutschland, ein Teil von Springer Nature 2020

==============================

■ Für die Forschung und die Forscherinnen und Forscher in Deutschland bringt die Coronavirus-Pandemie zwei Erfahrungen mit sich, die ganz unterschiedlich sind: Zum einen zeigt sich gerade jetzt, wie wichtig ihre Arbeit ist, bei der Suche nach Impfstoffen, Medikamenten und Therapien, aber auch als Orientierung und Beratung für Politik und Gesellschaft. Zum anderen führen die Pandemie und die zu ihrer Eindämmung beschlossenen Maßnahmen in vielen Forschungsprojekten zu Einschränkungen und Verzögerungen, wovon die eigentlichen Forschungsarbeiten betroffen sind, aber auch die in den Projekten Beschäftigten.

Damit sind auch bereits die beiden Herausforderungen adressiert, derer sich die Deutsche Forschungsgemeinschaft (DFG) als die größte Forschungsförderorganisation in Deutschland in diesen Wochen und Monaten angenommen hat: Einerseits galt und gilt es – bei größtmöglichem Schutz aller Beteiligten als oberste Priorität – die Förderung von mehr als 30.000 Forschungsprojekten aus allen Fachgebieten so reibungslos wie möglich fortzusetzen. Die zweite Herausforderung ist, der Forschung zu den vielfältigen Dimensionen der Pandemie weitere Impulse zu geben.

Auf beiden Feldern ist die DFG früh aktiv geworden. Seit Mitte März 2020 lenken die Mitarbeiterinnen und Mitarbeiter der DFG-Geschäftsstelle die Antragsbearbeitung und Finanzströme größtenteils vom Homeoffice aus; Begutachtungen finden per Telefon- und Videokonferenzen statt, und Förderentscheidungen werden im schriftlichen Verfahren getroffen. Dies ist bei allen Beteiligten mit großem Engagement und hoher Professionalität verbunden.

Darüber hinaus haben wir eine Reihe von Maßnahmen getroffen, um die finanziellen und zeitlichen Auswirkungen der Pandemie auf die Forschungen möglichst abzufedern. Dazu gehörten zunächst die kostenneutrale Verlängerung haushaltsjahrgebundener Projekte, ebenso Ausgleichs-, Überbrückungs- und Auslauffinanzierungen oder die Verlängerung von Ausschreibungen, Stipendien und Anstellungsverträgen von Doktorandinnen und Doktoranden. Mitte Mai 2020 nun haben wir zudem ein finanzielles Hilfspaket von mehr als 175 Millionen Euro aufgelegt. Mit ihm können unsere Geförderten auch zusätzliche Personal- und Sachmittel beantragen, wenn sie ihre Arbeiten infolge von Corona nicht in der geplanten Zeit und Produktivität durchführen können, was wir auch generell bei allen unseren Förderentscheidungen berücksichtigen werden. Dies alles bringt dringend benötigte Unterstützung und Sicherheit.

In fachlicher Hinsicht hat die DFG ebenfalls bereits früh neue Förderangebote für die Forschung zum Coronavirus geschaffen. Denn wie alle Infektionskrankheiten lässt sich auch diese Pandemie umso wirkungsvoller bekämpfen, je besser wir den Erreger und seine Auswirkungen wirklich verstehen. Dazu braucht es umfassende Forschungen, die mit langem Atem und erkenntnisgeleiteten Fragestellungen durchgeführt werden – und für deren Förderung die DFG in besonderer Weise steht.

Zusätzlich zu den bereits seit einer Reihe von Jahren geförderten Projekten zu Coronaviren und zur Infektiosität und genetischen Vielfalt von Viren haben wir deshalb Ende März 2020 eine fachübergreifende und thematisch bewusst breit angelegte Ausschreibung gestartet. Mit ihr sollen sowohl Forschungsvorhaben zu biologischen und medizinischen Grundlagen eines Erregers sowie zu präventiven Maßnahmen und therapeutischen Verfahren als auch zu psychologischen, gesellschaftlichen, kulturellen, rechtlichen, ethischen oder ökonomischen Implikationen gefördert werden. Mit einer solchen breit gefächerten Forschung wollen wir neben der Erforschung der aktuellen Pandemie zu generalisierbaren wissenschaftlichen Erkenntnissen beitragen, um künftig besser auf die vielfältigen Dimensionen von weltweiten Infektionswellen vorbereitet zu sein. Weitere, auch institutionelle Maßnahmen zur Stärkung gerade der Grundlagenforschung auf diesem Feld sind in Vorbereitung.

All dies verstehen wir nicht zuletzt als Teil weltweiter wissenschaftlicher Bemühungen zur Bekämpfung der Pandemie. Denn diese kann nur gelingen, wenn sie die globalen Zusammenhänge in den Blick nimmt und wenn das globale Wissen zusammengeführt wird. Wie in anderen Bereichen unserer Gesellschaft geht es auch in der Wissenschaft hier nicht mehr um Konkurrenz, sondern um enge Abstimmung, vorausschauendes Handeln, um Solidarität und den Schutz besonders Schutzbedürftiger.

Gewiss wird es auch nach dem Gipfel der Coronavirus-Pandemie einige Zeit dauern, bis die Forschungsprozesse wieder in normalen Bahnen verlaufen. Die DFG wird alles daransetzen, auch den zu erwartenden Übergangssituationen vorausschauend zu begegnen, sie aktiv mitzugestalten und der Forschung und allen an ihr Beteiligten die größtmögliche Unterstützung zu geben. ■

**Open Access:** Dieser Artikel wird unter der Creative Commons Namensnennung 4.0 International Lizenz veröffentlicht, welche die Nutzung, Vervielfältigung, Bearbeitung, Verbreitung und Wiedergabe in jeglichem Medium und Format erlaubt, sofern Sie den/die ursprünglichen Autor(en) und die Quelle ordnungsgemäß nennen, einen Link zur Creative Commons Lizenz beifügen und angeben, ob Änderungen vorgenommen wurden. Die in diesem Artikel enthaltenen Bilder und sonstiges Drittmaterial unterliegen ebenfalls der genannten Creative Commons Lizenz, sofern sich aus der Abbildungslegende nichts anderes ergibt. Sofern das betreffende Material nicht unter der genannten Creative Commons Lizenz steht und die betreffende Handlung nicht nach gesetzlichen Vorschriften erlaubt ist, ist für die oben aufgeführten Weiterverwendungen des Materials die Einwilligung des jeweiligen Rechteinhabers einzuholen. Weitere Details zur Lizenz entnehmen Sie bitte der Lizenzinformation auf http://creativecommons.org/licenses/by/4.0/deed.de.

**Korrespondenzadresse:**
Prof. Dr. Katja Becker
Deutsche Forschungsgemeinschaft (DFG)
Kennedyallee 40
D-53175 Bonn
praesidentin@dfg.de
www.dfg.de

„WIR HABEN EINE REIHE VON MAßNAHMEN GETROFFEN, UM DIE FINANZIELLEN UND ZEITLICHEN AUSWIRKUNGEN DER PANDEMIE AUF DIE FORSCHUNGEN MÖGLICHST ABZUFEDERN.“

Präsidentin der Deutschen Forschungsgemeinschaft, Professorin für Biochemie und Molekularbiologie an der Justus-Liebig-Universität Gießen

Funding Open Access funding provided by Projekt DEAL.

